# Gut bacterial tyrosine decarboxylases restrict levels of levodopa in the treatment of Parkinson’s disease

**DOI:** 10.1038/s41467-019-08294-y

**Published:** 2019-01-18

**Authors:** Sebastiaan P. van Kessel, Alexandra K. Frye, Ahmed O. El-Gendy, Maria Castejon, Ali Keshavarzian, Gertjan van Dijk, Sahar El Aidy

**Affiliations:** 1 Department of Molecular Immunology and Microbiology, Groningen Biomolecular Sciences and Biotechnology Institute (GBB), University of Groningen, Nijenborgh 7, 9747 AG Groningen, The Netherlands; 2 Division of Digestive Disease and Nutrition, Section of Gastroenterology, Department of Internal Medicine, Rush University Medical Center, 1725 W. Harrison, Suite 206, Chicago, Illinois 60612 USA; 3 Department of Behavioral Neuroscience, Groningen Institute for Evolutionary Life Sciences (GELIFES), University of Groningen, Nijenborgh 7, 9747 AG Groningen, The Netherlands; 4 Present Address: Faculty of Pharmacy, Department of Microbiology and Immunology, Beni-Suef University, Beni-Suef, 62514 Egypt

## Abstract

Human gut microbiota senses its environment and responds by releasing metabolites, some of which are key regulators of human health and disease. In this study, we characterize gut-associated bacteria in their ability to decarboxylate levodopa to dopamine via tyrosine decarboxylases. Bacterial tyrosine decarboxylases efficiently convert levodopa to dopamine, even in the presence of tyrosine, a competitive substrate, or inhibitors of human decarboxylase. In situ levels of levodopa are compromised by high abundance of gut bacterial tyrosine decarboxylase in patients with Parkinson’s disease. Finally, the higher relative abundance of bacterial tyrosine decarboxylases at the site of levodopa absorption, proximal small intestine, had a significant impact on levels of levodopa in the plasma of rats. Our results highlight the role of microbial metabolism in drug availability, and specifically, that abundance of bacterial tyrosine decarboxylase in the proximal small intestine can explain the increased dosage regimen of levodopa treatment in Parkinson’s disease patients.

## Abstract (web summary)

The gut microbiota can impact the bioavailability of therapeutic drugs. Here, the authors show that bacterial tyrosine decarboxylases (TDC) decrease the levels of levodopa, the primary treatment in Parkinson’s disease, by conversion to dopamine, and suggest TDC as a potential predictive biomarker for treatment.

## Introduction

Gut bacteria interfere with effectiveness of drug treatment. The complex bacterial communities inhabiting the mammalian gut have a significant impact on the health of their host^1^. Numerous reports indicate that intestinal microbiota, and in particular its metabolic products, have a crucial effect on various health and diseased states. Host immune system and brain development, metabolism, behavior, stress and pain response all have been reported to be associated with microbiota disturbances^2–6^. In addition, it is becoming increasingly clear that gut microbiota can interfere with the modulation of drug efficacy^7,8^.

Parkinson’s disease (PD), the second most common neurodegenerative disorder, affecting 1% of the global population over the age of 60, and has recently been correlated with alterations in microbial gut composition^9–11^. The primary treatment of PD is levodopa (L-3,4-dihydroxyphenylalanine or L-DOPA) in combination of an aromatic amino acid decarboxylase inhibitor (primarily carbidopa)^12^. However, the bioavailability of levodopa/ decarboxylase inhibitor, required to ensure sufficient amounts of dopamine will reach the brain^13^, varies significantly among PD patients. Because of this, levodopa/ decarboxylase inhibitor is ineffective in a subset of patients, and its efficacy decreases over time of treatment, necessitating more frequent drug doses, ranging from 3 to 8-10 tablets/day with higher risk of dyskinesia and other side effects^14^. A major challenge in the clinic is an early diagnosis of motor response fluctuation (timing of movement‐related potentials) and decreased levodopa/ decarboxylase inhibitor efficacy to determine optimal dosage for individual patients and during disease progression. What remains to be clarified is whether inter-individual variations in gut microbiota composition and functionality play a causative role in motor response fluctuation in PD patients requiring higher daily levodopa/decarboxylase inhibitor treatment dosage regimen.

In fact, it had been shown that large intestinal microbiota could mainly dehydroxylate levodopa as detected in urine and cecal content of conventional rats^15^. However, these results do not explain a possible role of gut microbiota in the increased dosage regimen of levodopa/decarboxylase inhibitor treatment in PD patients because the primary site of levodopa absorption is the proximal small intestine (jejunum)^16^.

Several amino acid decarboxylases have been identified in bacteria. Tyrosine decarboxylase (TDC) genes (*tdc*) have especially been encoded in the genome of several bacterial species in the genera *Lactobacillus* and *Enterococcus*^17,18^. Though TDC is named for its capacity to decarboxylate L-tyrosine into tyramine, it might also have the ability to decarboxylate levodopa to produce dopamine due to the high similarity of the chemical structures of these substrates. This implies that TDC activity of the gut microbiota might interfere with levodopa/decarboxylase inhibitor availability, thus the treatment of PD patients.

The aim of the present study is to parse out the effect of levodopa metabolizing bacteria, particularly in the jejunum, where levodopa is absorbed. Initially, we established TDC present in small intestinal bacteria efficiently converted levodopa to dopamine, confirming their capacity to influence the in situ levels of the primary treatment of PD patients. We show that higher relative abundance of bacterial *tdc* gene in stool samples of PD patients positively correlates with higher daily levodopa/carbidopa dosage requirement and duration of disease. We further confirm our findings in rats orally administered levodopa/carbidopa, illustrating that levodopa levels in plasma negatively correlate with the abundance of bacterial *tdc* gene in the jejunum.

## Results

### Upper small intestinal bacteria convert levodopa to dopamine

**Fig. 1:**
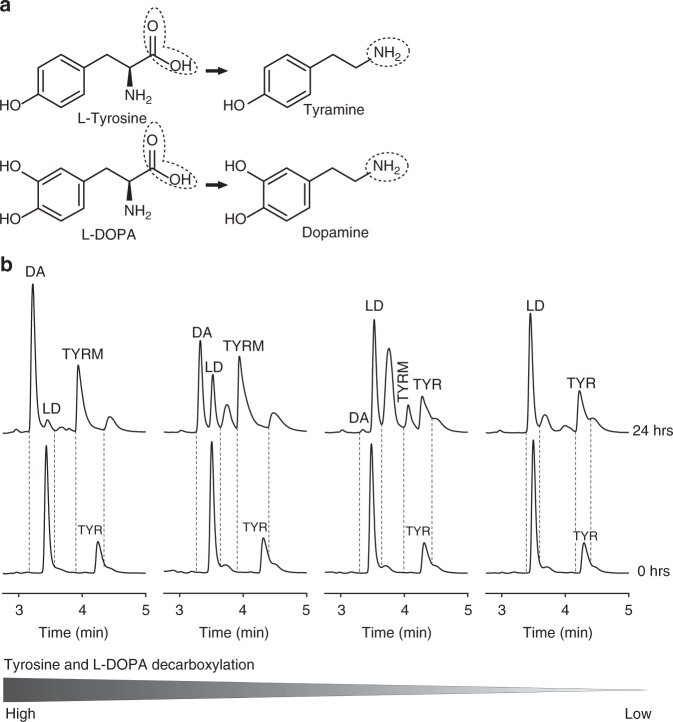
Bacteria in jejunal content decarboxylate levodopa to dopamine coinciding with their production of tyramine ex vivo. **a** Decarboxylation reaction for tyrosine and levodopa. **b** From left to right coinciding bacterial conversion of tyrosine (TYR) to tyramine (TYRM) and 1 mM of supplemented levodopa (LD) to dopamine (DA) during 24 h of incubation of jejunal content. The jejunal contents are from four different rats ranked form left to right based on the decarboxylation levels of tyrosine and levodopa, showing that tyrosine decarboxylation is coinciding with levodopa decarboxylation

To determine whether jejunal microbiota maintain the ability to metabolize levodopa, luminal samples from the entire jejunum of wild-type Groningen rats housed in different cages were incubated in vitro with levodopa and analyzed by High-Performance Liquid Chromatography with Electrochemical Detection (HPLC-ED). Chromatograms revealed that levodopa decarboxylation to dopamine coincide with the conversion of tyrosine to tyramine (Fig. 1a). Ranking the chromatograms from high to low decarboxylation of levodopa and tyrosine, shows that only when tyrosine is decarboxylated, dopamine is produced (Fig. 1b). No other metabolites were detected in the treated samples, except of few unknown peaks, which were also present in the control samples, thus are not products of bacterial metabolism of levodopa. In addition, no dopamine production was observed in control samples (Supplementary Fig. 1). Of note, no basal levels of levodopa were detected in the measured samples by HPLC. Taken together, the results suggest that bacterial TDC is involved in levodopa conversion into dopamine, which may, in turn, interfere with levodopa uptake in the proximal small intestine.

### Levodopa decarboxylation by bacterial TDC

**Fig. 2:**
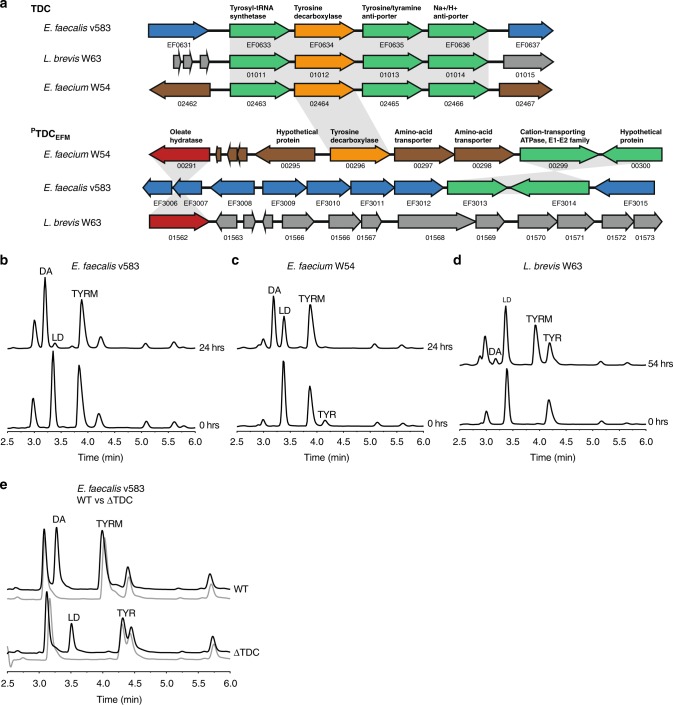
Gut bacteria harboring tyrosine decarboxylases are responsible for levodopa decarboxylation. **a** Aligned genomes of *E. faecium*, *E. faecalis*, and *L. brevis*. The conserved *tdc*-operon is depicted with *tdc* gene in orange. Overnight cultures of **b** *E. faecalis* v583, **c** *E. faecium* W54, and **d** *L. brevis* W63 incubated anaerobically at 37 °C with 100 µM of levodopa (LD). **e** Overnight cultures of EFS^WT^ and EFS^ΔTDC^ incubated anaerobically at 37 °C with 100 μM levodopa (black line) compared to control (gray line) where no levodopa was added. Curves represent one example of three biological replicates

The coinciding tyrosine and levodopa decarboxylation observed in the luminal content of jejunum was the basis of our hypothesis that TDC is the enzyme involved in both conversions. Species of the genera *Lactobacillus* and *Enterococcus* have been reported to harbor this enzyme^17,19^. To identify whether the genome of other (small intestinal) gut bacteria also encode *tdc*, the TDC protein sequence (EOT87933) from *Enterococcus faecalis* v583 was used as a query to search the US National Institutes of Health Human Microbiome Project (HMP) protein database. This analysis exclusively identified TDC proteins in species belonging to the bacilli class, including more than 50 *Enterococcus* strains (mainly *E. faecium* and *E. faecalis*) and several *Lactobacillus* and *Staphylococcus* species (Supplementary Fig. 2a). Next, we aligned the genome of *E. faecalis* v583 with two gut bacterial isolates, *E. faecium* W54, and *L. brevis* W63, illustrating the conservation of the *tdc*-operon among these species (Fig. 2a). Intriguingly, analysis of *E. faecium* genomes revealed that this species encodes a second, paralogous *tdc* gene (^P^TDC_EFM_) that did not align with the conserved *tdc*-operon and was absent from the other species (Fig. 2a, Supplementary Figs. 2a and 6).

To support our in silico data, a comprehensive screening of *E. faecalis* v583, *E. faecium* W54, and *L. brevis* W63 and 77 additional clinical and human isolates of *Enterococcus*, including clinical isolates and strains from healthy subjects, was performed. All enterococcal isolates and *L. brevis* were able to convert tyrosine and levodopa into tyramine and dopamine, respectively (Fig. 2b–d, Supplementary Table 1). Notably, our HPLC-ED analysis revealed considerable variability among the tested strains with regard to their efficiency to decarboxylate levodopa. *E. faecium* and *E. faecalis* were drastically more efficient at converting levodopa to dopamine, compared to *L. brevis*. Growing *L. brevis* in different growth media did not change the levodopa decarboxylation efficacy (Supplementary Fig. 2b, c). To eliminate the possibility that other bacterial amino acid decarboxylases are involved in levodopa conversion observed in the jejunal content we expanded our screening to include live bacterial species harboring PLP-dependent amino acid decarboxylases previously identified by Williams et al.^20^. None of the tested bacterial strains encoding different amino acid decarboxylases could decarboxylate levodopa (Supplementary Fig. 2d–g, Supplementary Table 2).

To verify that the TDC is solely responsible for levodopa decarboxylation in *Enterococcus*, wild-type *E. faecalis* v583 (EFS^WT^) was compared with a mutant strain (EFS^ΔTDC^)^17^. Overnight incubation of EFS^WT^ and EFS^ΔTDC^ bacterial cells with levodopa resulted in production of dopamine in the supernatant of EFS^WT^ but not EFS^ΔTDC^ (Fig. 2e), confirming the pivotal role of this gene in this conversion. Collectively, results show that TDC is encoded on genomes of gut bacterial species known to dominate the proximal small intestine and that this enzyme is exclusively responsible for converting levodopa to dopamine by these bacteria, although the efficiency of that conversion displays considerable species-dependent variability.

### Tyrosine abundance does not prevent levodopa decarboxylation

**Fig. 3:**
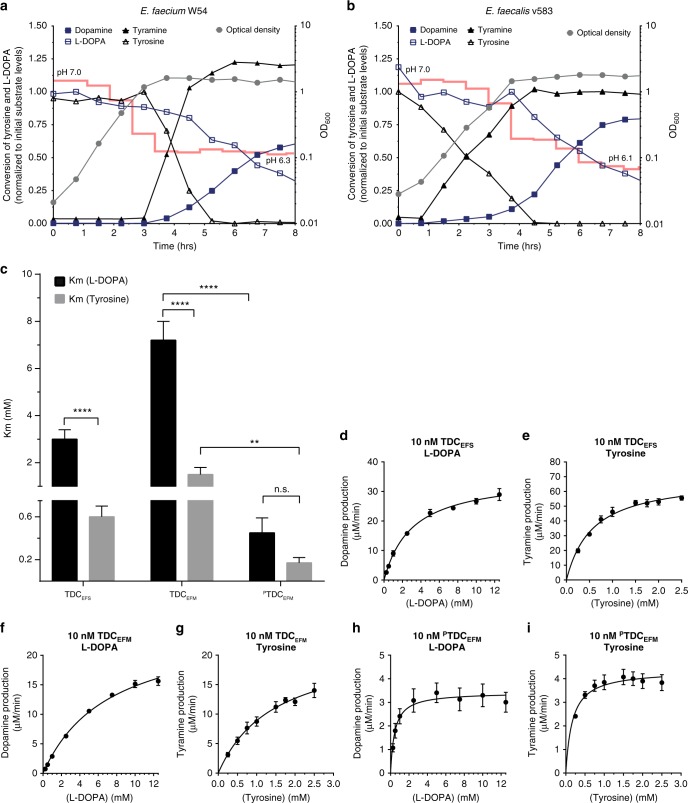
Enterococci decarboxylate levodopa in presence of tyrosine despite higher affinity for tyrosine in vitro. Growth curve (gray circle, right *Y*-axis) of *E. faecium* W54 (**a**) and *E. faecalis* (**b**) plotted together with levodopa (open square), dopamine (closed square), tyrosine (open triangle), and tyramine (closed triangle) levels (left *Y*-axis). Concentrations of product and substrate were normalized to the initial levels of the corresponding substrate (100 µM supplemented levodopa and ~500 µM tyrosine present in the medium). pH of the culture is indicated over time as a red line. **c** Substrate affinity (Km) for levodopa and tyrosine for purified tyrosine decarboxylases from *E. faecalis* v583 (TDC_EFS_), *E. faecium* W54 (TDC_EFM_, ^P^TDC_EFM_). **d**–**i** Michaelis–Menten kinetic curves for levodopa and tyrosine as substrate for TDC_EFS_ (**d**, **e**), TDC_EFM_ (**f**, **g**), and ^P^TDC_EFM_ (**h**, **i**). Reactions were performed in triplicate using levodopa concentrations ranging from 0.5 to 12.5 mM and tyrosine concentrations ranging from 0.25 to 2.5 mM. The enzyme kinetic parameters were calculated using nonlinear Michaelis–Menten regression model. Error bars represent the SEM and significance was tested using 2-way-Anova, Fisher LSD test, (*p < 0.02; **p < 0.01; ****<0.0001)

To test whether the availability of the primary substrate for bacterial TDC (i.e., tyrosine) could inhibit the uptake and decarboxylation of levodopa, the growth, metabolites, and pH that was previously shown to affect the expression of *tdc*^17^, of *E. faecium* W54 and *E. faecalis* v583 were analyzed over time. A volume of 100 µM levodopa was added to the bacterial cultures, whereas ~500 µM tyrosine was present in the growth media, which corresponds to the levels of tyrosine found in the jejunum^21^. Remarkably, levodopa and tyrosine were converted simultaneously, even in the presence of these excess levels of tyrosine (1:5 levodopa to tyrosine), albeit at a slower conversion rate for levodopa (Fig. 3a, b). Notably, the decarboxylation reaction appeared operational throughout the exponential phase of growth for *E. faecalis*, whereas it is only observed in *E. faecium* when this bacterium entered the stationary phase of growth, suggesting differential regulation of the *tdc* gene expression in these species.

To further characterize the substrate specificity and kinetic parameters of the bacterial TDCs, *tdc* genes from *E. faecalis* v583 (TDC_EFS_) and *E. faecium* W54 (TDC_EFM_ and ^P^TDC_EFM_) were expressed in *Escherichia coli* BL21 (DE3) and then purified. Michaelis–Menten kinetics indicated each of the studied enzymes had a significantly higher affinity (*K*_m_) (Fig. 3c–i) and catalytic efficiency (*K*_cat_/*K*_m_) for tyrosine than for levodopa (Table 1). Despite the differential substrate affinity, our findings illustrate that high levels of tyrosine do not prevent the decarboxylation of levodopa in batch culture.

**Table 1:** Michaelis–Menten kinetic parameters

Levodopa	pH 5.0	pH 5.0	pH 4.5	pH 7.4
	TDC_EFS_	TDC_EFM_	^P^TDC_EFM_	DDC
[E] (nM)	10	10	10	10
Km (mM)	3 ± 0.4	7.2 ± 0.8	0.4 ± 0.1	0.1 ± 0.01
Vmax (µM/min)	35.3 ± 1.4	25.5 ± 1.3	3.4 ± 0.2	1.4 ± 0.03
Kcat (min^−1^)	3531 ± 137	2549 ± 133	342.4 ± 21	136.9 ± 3
Kcat/Km (min^−1^/mM^−1^)	1160	352	764	1567
R^2^	0.978	0.99	0.621	0.962
Tyrosine	pH 5.0	pH 5.0	pH 4.5	
	TDC_EFS_	TDC_EFM_	^P^TDC_EFM_	
[E] (nM)	10	10	10	
Km (mM)	0.6 ± 0.1	1.5 ± 0.3	0.2 ± 0.05	
Vmax (µM/min)	69.6 ± 2.9	22 ± 2.5	4.4 ± 0.2	
Kcat (min^−1^)	6963 ± 288	2204 ± 247	435.6 ± 19.2	
Kcat/Km (min^−1^/mM^−1^)	12216	1493	2558	
R^2^	0.928	0.902	0.589	

Enzyme kinetic parameters were determined by Michaelis–Menten nonlinear regression model for levodopa and tyrosine as substrates. ± indicates the standard error

### Carbidopa does not inhibit bacterial decarboxylases

**Fig. 4:**
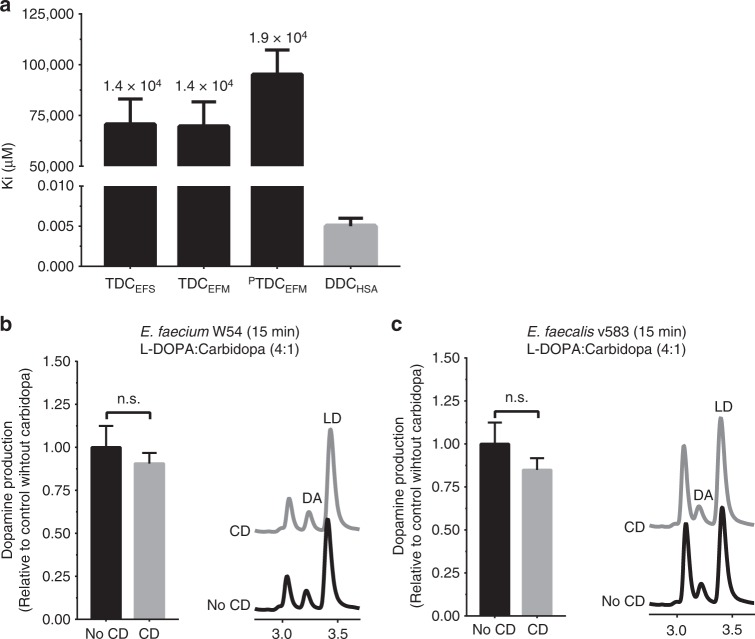
Human DOPA decarboxylase inhibitor, carbidopa, does not inhibit bacterial tyrosine decarboxylases. **a** Inhibitory constants (Ki) of bacterial decarboxylases (black) and human DOPA decarboxylase (gray), with fold-difference between bacterial and human decarboxylase displayed on top of the bars. Quantitative comparison of dopamine (DA) production by *E. faecium* W54, **b** and *E. faecalis* v583, **c** at stationary phase after 15 min, with representative HPLC-ED curve. Bacterial cultures (*n* = 3) were incubated with 100 µM levodopa (LD) or a 4:1 mixture (in weight) of levodopa and carbidopa (CD) (100 µM levodopa and 21.7 µM carbidopa). Error bars represent SEM (**a**) or SD (**b**, **c**) and significance was tested using a parametric unpaired *T*-test

To assess the extent to which human DOPA decarboxylase inhibitors could affect bacterial decarboxylases, three human DOPA decarboxylase inhibitors (carbidopa, benserazide, and methyldopa) were tested on purified bacterial TDCs and on the corresponding bacterial batch cultures. Comparison of the inhibitory constants (K_i_^TDC^/K_i_^DDC^) demonstrates carbidopa to be a 1.4–1.9 × 10^4^ times more potent inhibitor of human DOPA decarboxylase than bacterial TDCs (Fig. 4a, Supplementary Fig. 3; Supplementary Table 3). This is best illustrated by the observation that levodopa conversion by *E. faecium* W54 and *E. faecalis* v583 batch cultures (OD_600_ = ~2.0) was unaffected by co-incubation with carbidopa (equimolar or 4-fold carbidopa relative to levodopa) (Fig. 4b, c, Supplementary Fig. 4a). Analogously, benserazide and methyldopa did not inhibit the levodopa decarboxylation activity in *E. faecalis* or *E. faecium* (Supplementary Fig. 4b, c).

These findings demonstrate the commonly applied inhibitors of human DOPA decarboxylase in levodopa combination therapy do not inhibit bacterial TDC dependent levodopa conversion, implying levodopa/carbidopa (levodopa) combination therapy for PD patients would not affect the efficacy of levodopa in situ by small intestinal bacteria.

### PD dosage regimen correlates with *tdc* gene abundance

**Fig. 5:**
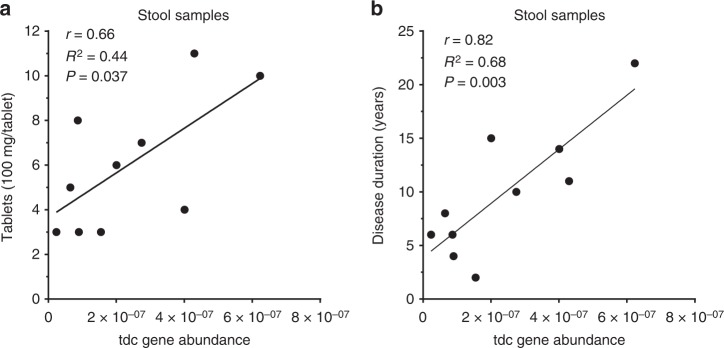
Tyrosine decarboxylase gene abundance correlates with daily levodopa dose and disease duration in fecal samples of Parkinson’s disease patients. **a** Scatter plot of *tdc* gene abundance measured by qPCR in fecal samples of PD patients (*n* = 10) versus daily levodopa/carbidopa dosage fitted with linear regression model. **b** Scatter plot of *tdc* gene abundance from the same samples versus disease duration fitted with a linear regression model. Pearson’s *r* analysis was used to determine significant correlations between tyrosine decarboxylase gene abundance and dosage (*r* = 0.66, *R*^2^ = 0.44, *P* value = 0.037) or disease duration (*r* = 0.82, *R*^2^ = 0.68, *P* value = 0.003)

To determine whether the increased dosage regimen of levodopa treatment in PD patients could be attributed to the abundance of *tdc* genes in the gut microbiota, fecal samples were collected from male and female PD patients (Supplementary Table 4) on different doses of levodopa/carbidopa treatment (ranging from 300 up to 1100 mg levodopa per day). *tdc* gene-specific primers were used to quantify its relative abundance within the gut microbiota by qPCR and results were normalized to 16S rRNA gene to correct for difference in total bacterial counts among the stool samples (Supplementary Fig. 5). Remarkably, Pearson *r* correlation analyses showed a strong positive correlation (*r* = 0.66, *R*^2^ = 0.44, *p* value = 0.037) between bacterial *tdc* gene relative abundance and levodopa/carbidopa treatment dose (Fig. 5a), as well as with the duration of disease (Fig. 5b, Supplementary Table 5). Collectively, the selective prevalence of *tdc* encoding genes in the genomes of signature microbes of the small intestine microbiota supports the notion that the results obtained from fecal samples are a valid representation of *tdc* gene abundance in the small intestinal microbiota. Moreover, the significant correlation of the relative *tdc* abundance in the fecal microbiota and the required levodopa/carbidopa dosage strongly supports a role for bacterial TDC in levodopa/carbidopa efficacy.

At this stage, it is not demonstrated whether the relative abundance of *tdc* in fecal samples reflects its abundance in the proximal small intestine. This is of particular importance because levodopa is absorbed in the proximal small intestine, and reduction in its bioavailability by bacterial TDC activity in the context of PD patients’ medication regimens would only be relevant in that intestinal region.

### Higher *tdc* gene abundance restricts levodopa level in plasma

**Fig. 6:**
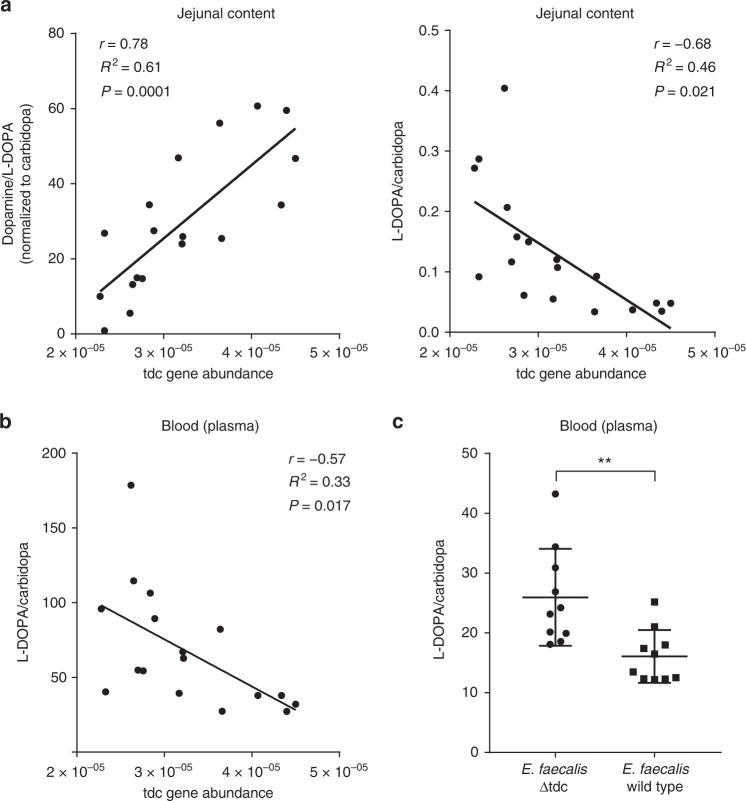
Luminal and plasma levels of levodopa are compromised by higher abundance of tyrosine decarboxylase gene in the small intestine of rats. Scatter plot of *tdc* gene abundance measured by qPCR in jejunal content of wild-type Groningen rats (*n* = 18) orally supplied with levodopa/carbidopa mixture (4:1) versus **a** the dopamine: levodopa/carbidopa levels in the jejunal content, the levodopa/carbidopa levels in the jejunal content, **b** or the levodopa/carbidopa levels in the plasma, fitted with a linear regression model. Intake of levodopa/carbidopa was corrected by using carbidopa as an internal standard. Pearson’s *r* correlation was used to determine significant correlations between *tdc* abundance and jejunal dopamine levels (*r* = 0.78, *R*^2^ = 0.61, *P* value = 0.0001), jejunal levodopa/carbidopa levels (*r* = −0.68, *R*^2^ = 0.46  *P* value = 0.021), or plasma levodopa/carbidopa levels (*r* = −0.57, *R*^2^ = 0.33, *P* value = 0.017). No levodopa/carbidopa, dopamine, or DOPAC were detected in the control group (*n* = 5). **c** Significant difference in plasma levels of levodopa/carbidopa orally supplied to rats after treatment with EFS^WT^ (*n* = 10) or EFS^ΔTDC^ (*n* = 10). Significance was tested using parametric unpaired *T*-test (**p < 0.01)

To further consolidate the concept that *tdc* gene abundance in proximal small intestinal microbiota affects peripheral levels of levodopa/carbidopa in blood and dopamine: levodopa/carbidopa ratio in the jejunal luminal content, male wild-type Groningen rats (*n* = 18) rats were orally administered 15 mg levodopa/3.75 mg carbidopa per kg of body weight and sacrificed after 15 min (point of maximal levodopa bioavailability in rats^22^). Plasma levels of levodopa/carbidopa and its metabolite dopamine were measured by HPLC-ED, while relative abundance of the *tdc* gene within the small intestinal microbiota was quantified by gene-specific qPCR (Supplementary Fig. 5). Strikingly, Pearson *r* correlation analyses showed that the ratio between dopamine and levodopa/carbidopa levels in the proximal jejunal content positively correlated with *tdc* gene abundance (*r* = 0.78, *R*^2^ = 0.61, *P* value = 0.0001), whereas the levodopa/carbidopa concentration in the proximal jejunal content negatively correlated with the abundance of the *tdc* gene (*r* = −0.68, *R*^2^ = 0.46, *P* value = 0.021) (Fig. 6a). Moreover, plasma levels of levodopa/carbidopa displayed a strong negative correlation (*r* = −0.57, *R*^2^ = 0.33, *P* value = 0.017) with the relative abundance of the *tdc* gene (Fig. 6b). No basal levels of levodopa were detected in the measured samples by HPLC-ED.

To further support this correlation, plasma levels of levodopa/carbidopa from rats treated with EFS^WT^ (*n* = 10) or EFS^ΔTDC^ (*n* = 10) cells were determined after oral administration with levodopa/carbidopa mixture (4:1). Rats treated with EFS^WT^ showed significant lower levels (*P* value < 0.01) of levodopa/carbidopa in their plasma compared to rats treated with EFS^ΔTDC^ (Fig. 6c). Collectively, these findings clearly show that levodopa/carbidopa uptake by the host is compromised by higher abundance of gut bacteria encoding for *tdc* genes in the upper region of the small intestine.

## Discussion

Our observation that the jejunal microbiota are able to convert levodopa to dopamine (Fig. 1) was the basis of investigating the role of levodopa metabolizing bacteria in the context of the disparity in increased dosage regimen of levodopa/carbidopa treatment in a subset of PD patients (Fig. 5) and the accompanying adverse side effects^23^. This study identifies a significant factor to explain the motor response (timing of movement‐related potentials) fluctuations observed in PD patients requiring frequent levodopa/decarboxylase inhibitor administration.

Our primary outcome is that levodopa decarboxylation by small intestinal bacteria, in particular, members of bacilli, including the genera *Enterococcus* and *Lactobacillus*, which were previously identified as the predominant residents of the small intestine^24,25^, would drastically reduce the levels of levodopa/decarboxylase inhibitor in the body, and thereby contribute to the observed higher dosages required in a subset of PD patients. Previously, reduced levodopa availability has been associated with *Helicobacter pylori* positive PD patients, which was explained by the observation that *H. pylori* could bind levodopa in vitro via surface adhesins^8^. However, this explanation is valid only for a small population of the PD patients, who suffer from stomach ulcers and thus have high abundance of *H. pylori*.

**Fig. 7:**
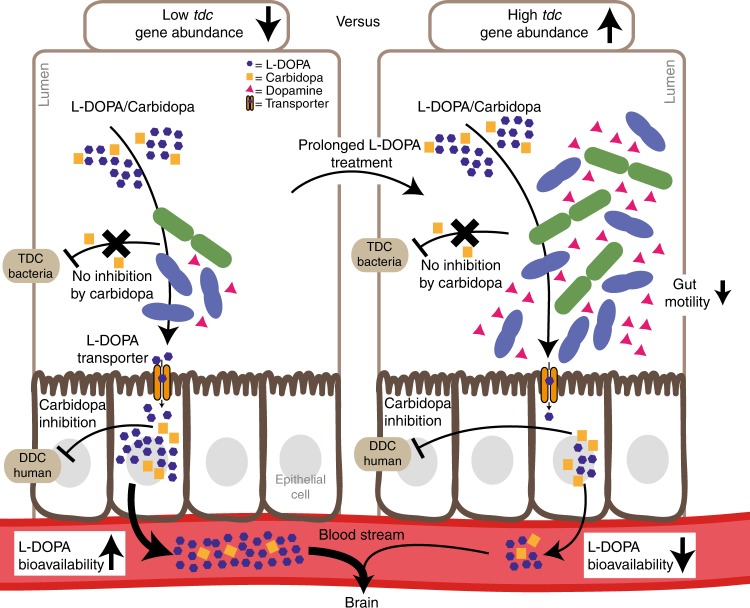
Higher abundance of tyrosine decarboxylase can explain increased levodopa administration requirement in Parkinson’s disease patients. A model representing two opposing situations, in which the proximal small intestine is colonized by low (left) or high abundance of tyrosine decarboxylase-encoding bacteria. The latter could result from or lead to increased individual L-DOPA dosage intake

The impaired intestinal motility frequently observed in PD patients^26^ could also result from altered levels of dopamine, the conversion product of bacterial *tdc* metabolism of levodopa^27^ but has been also associated with small intestinal bacterial overgrowth^28^, and worsening of motor response fluctuations thus requiring higher dosage frequency of levodopa/decarboxylase inhibitor treatment^29^. Moreover, the decreasing efficacy of levodopa treatment observed in PD patients might be explained by the overgrowth of small intestinal bacteria that metabolize levodopa resulting from proton pump inhibitors^30–32^^,^ for treatment of gastrointestinal symptoms. In particular, *Enterococcus* has been reported to dominate in proton pump inhibitors’ induced small intestinal bacterial overgrowth^33^. Altogether, these factors will enhance a state of small intestinal bacterial overgrowth, and perpetuating a vicious circle leading to increased levodopa/decarboxylase inhibitor dosage requirement in a subset of PD patients (Fig. 7). Finally, it is likely that prolonged levodopa/ decarboxylase inhibitor administration favors growth of *tdc* expressing bacteria in the proximal small intestine, resulting in higher levels of *tdc* further lowering the efficacy of levodopa. In fact, it has been shown that the fitness of *E. faecalis* v583 in low pH depends on the *tdc*-operon^17^, indicating long-term exposure to levodopa could contribute to selection for overgrowth of *tdc* encoding bacteria in vivo as supported by the positive correlation with *tdc* gene abundance observed in human stool samples (Fig. 5b). This would explain the fluctuating motor response and subsequent increased levodopa/decarboxylase inhibitor dosage regimen thus severity of its adverse effects, such as dyskinesia during prolonged disease treatment^34^.

While our further investigation into the kinetics of both bacterial and human decarboxylases support the effectiveness of carbidopa to inhibit the human DOPA decarboxylase, it also shows that the same drug fails to inhibit levodopa decarboxylation by bacterial TDC, probably due to the presence of an extra hydroxyl group on the benzene ring of carbidopa (Fig. 4, Supplementary Fig. 3) or ineffective transport of the inhibitor inside the bacterial cell. This suggests a better equilibration of levodopa treatment between patients could potentially be achieved by co-administration of an effective TDC inhibitor that targets both human and bacterial decarboxylases. Alternatively, we are currently evaluating regulation of *tdc* gene expression to help avoid the need for high levodopa dosing, thus minimizing its adverse side effects.

Notably, a few *Enterococcus* strains that harbor the *tdc* gene are marked as probiotics. The use of such strains as dietary supplements should be recognized in case of PD patients. More generally, our data support the increasing interest in the impact that gut microbiota metabolism may have on medical treatment and diet.

Collectively, our data show that levodopa conversion by bacterial TDC in the small intestine should be considered as a significant explanatory factor for the increased levodopa/carbidopa dosage regimen required in a subset of PD patients. Although the data from PD patients are tentative due to small number of samples, this study strongly suggests these bacteria or their encoded *tdc* gene may potentially serve as a predictive biomarker to stratify PD patients for efficacy of levodopa treatment as supported by the significant (*r* = 0.66) correlation observed between the relative abundance of bacterial *tdc* genes in stool samples of patients and number of levodopa/carbidopa tablets required to treat individual PD patients (Fig. 5). To overcome the limitation of the small number of samples from PD patients in this study, we are currently validating the development of such a simple cost-effective novel biomarker for optimal dosage of levodopa/carbidopa and to prevent side effects in a large longitudinal cohort of newly diagnosed PD patients, who are followed over long periods of time.

## Methods

### Human fecal samples from patients with Parkinson’s disease

Fecal samples from patients diagnosed with Parkinson’s disease (*n* = 10) on variable doses (300–1100 mg levodopa per day) of levodopa/carbidopa treatment were acquired from the Movement Disorder Center at Rush University Medical Center, Chicago, Illinois, USA. Patients’ characteristics were published previously^35^ (more details are provided in Supplementary Table 4). Solid fecal samples were collected in anaerobic fecal bags and kept sealed in a cold environment until brought to the hospital where they were immediately stored at −80 °C until analysis.

### Rats

All animal procedures were approved by the Groningen University Committee of Animal experiments (approval number: AVD1050020184844), and were performed in adherence to the NIH Guide for the Care and Use of Laboratory Animals.

Twenty-five male wild-type Groningen rats (Groningen breed, male, age 18–24 weeks) housed 4–5 animals/cage had ad libitum access to water and food (RMH-B, AB Diets; Woerden, the Netherlands) in a temperature (21 ± 1 °C) and humidity-controlled room (45–60% relative humidity), with a 12 h light/dark cycle (lights off at 1:00 p.m.). These outbred rats are very frequently used in behavioral studies^36^ due to the high inter-individual variation (also in their microbiota composition), thus resembling, to some extent, the human inter-individual variation. On ten occasions over a period of three weeks, rats were taken from their social housing cage between circadian times 6 and 16.5, and put in an individual training cage (L × W × H = 25 × 25 × 40 cm) with a layer of their own sawdust without food and water. Ten minutes after transfer to these cages, rats were offered a drinking pipette in their cages with a 2.5 ml saccharine solution (1.5 g/L, 12476, Sigma). Over the course of training, all rats learned to drink the saccharine solution avidly. On the 11^th^ occasion, the saccharine solution was used as vehicle for the levodopa/carbidopa mixture (15/3.75 mg/kg), which all rats drank within 15 s. Fifteen minutes after drinking the latter mixture (maximum bioavailability time point of levodopa in blood as previously described^22^, the rats were anesthetized with isoflurane and sacrificed. Blood was withdrawn by heart puncture and placed in tubes pre-coated with 5 mM EDTA. The collected blood samples were centrifuged at 1500× *g* for 10 min at 4 °C and the plasma was stored at −80 °C prior to levodopa, dopamine, and DOPAC extraction. Luminal contents were harvested from the entire rat jejunum by gentle pressing and were snap frozen in liquid N_2_, stored at −80 °C until used for qPCR, and extraction of levodopa and its metabolites. The jejunum was distinguished from ileum by length (the intestinal tubes starting at 5 cm from stomach to cecum was divided into two; the proximal part was considered jejunum) Oral administration (by drinking, with saccharine as vehicle) of levodopa was corrected for by using carbidopa as an internal standard to correct for intake. Further, five rats were used as control and were administered a saccharine only solution (vehicle) to check for basal levels of levodopa, dopamine, and DOPAC levels or background HPLC-peaks. Jejunal content of control rats was used in ex vivo fermentation experiments (see incubation experiments of jejunal content section).

### Treatment with EFS^WT^ and EFS^ΔTDC^ bacteria

Rats (*n* = 20) were treated orally with 200 mg/kg body weight Rifaximin (R9904, Sigma) for five consecutive days as previously shown^29^. Subsequently, the rats were treated orally with 10^10^–10^11^ CFU wild type (*n* = 10) or Δ*tdc* (*n* = 10) *E. faecalis* v583 cells (EFS^WT^ and EFS^ΔTDC^ respectively) for five other consecutive days. One day following the bacterial treatment, the rats were orally supplied with levodopa/carbidopa mixture (4:1) as described above.

### Bacteria

*Escherichia coli* DH5a or BL21 were routinely grown aerobically in Luria-Broth (LB) at 37 °C degrees with continuous agitation. Other strains listed in Supplementary Table 6 were grown anaerobically (10% H_2_, 10% CO_2_, 80% N_2_) in a Don Whitley Scientific DG250 Workstation (LA Biosystems, Waalwijk, The Netherlands) at 37 °C in an enriched beef broth based on SHIME medium^37^ (Supplementary Table 7). Bacteria were inoculated from −80 °C stocks and grown overnight. Before the experiment, cultures were diluted 1:100 in fresh medium from overnight cultures. Levodopa (D9628, Sigma, The Netherlands), carbidopa (C1335, Sigma), benserazide (B7283, Sigma), or methyldopa (857416, Sigma) were supplemented during the lag or stationary phase depending on the experiment. Growth was followed by measuring the optical density (OD) at 600 nM in a spectrophotometer (UV1600PC, VWR International, Leuven, Belgium).

### Cloning and heterologous gene expression

The human DOPA decarboxylase gene cloned in pET15b was ordered from GenScript (Piscataway, USA) (Supplementary Table 6). TDC-encoding genes from *E. faecalis* v583 (TDC_EFS,_ accession: EOT87933), *E. faecium* W54 (TDC_EFM_, accession: MH358385; ^P^TDC_EFM_, accession: MH358384) were amplified using Phusion High-fidelity DNA polymerase and primers listed in Supplementary Table 8. All amplified genes were cloned in pET15b, resulting in pSK18, pSK11, and pSK22, respectively (Supplementary Table 6). Plasmids were maintained in *E. coli* DH5α and verified by Sanger sequencing before transformation to *E. coli* BL21 (DE3). Overnight cultures were diluted 1:50 in fresh LB medium with the appropriate antibiotic and grown to OD_600_ = 0.7–0.8. Protein translation was induced with 1 mM Isopropyl β-D-1-thiogalactopyranoside (IPTG, 11411446001, Roche Diagnostics) and cultures were incubated overnight at 18 °C. The cells were washed with 1/5th of 1 × ice-cold PBS and stored at −80 °C or directly used for protein isolation. Cell pellets were thawed on ice and resuspended in 1/50th of buffer A (300 mM NaCl; 10 mM imidazole; 50 mM KPO4, pH 7.5) containing 0.2 mg/mL lysozyme (105281, Merck) and 2 µg/mL DNAse (11284932001, Roche Diagnostics), and incubated for at least 10 min on ice before sonication (10 cycles of 15 s with 30 s cooling at 8 microns amplitude) using Soniprep-150 plus (Beun de Ronde, Abcoude, The Netherlands). Cell debris were removed by centrifugation at 20,000 × *g* for 20 min at 4 °C. The 6 × his-tagged proteins were purified using a nickel-nitrilotriacetic acid (Ni-NTA) agarose matrix (30250, Qiagen). Cell-free extracts were loaded on 0.5 ml Ni-NTA matrixes and incubated on a roller shaker for 2 h at 4 °C. The Ni-NTA matrix was washed three times with 1.5 ml buffer B (300 mM NaCl; 20 mM imidazole; 50 mM KPO4, pH 7.5) before elution with buffer C (300 mM NaCl; 250 mM imidazole; 50 mM KPO4, pH 7.5). Imidazole was removed from purified protein fractions using Amicon Ultra centrifugal filters (UFC505024, Merck) and washed three times and reconstituted in buffer D (50 mM Tris-HCL; 300 mM NaCl; pH 7.5) for TDC_EFS_, and TDC_EFM,_ buffer E (100 mM KPO4; pH 7.4) for ^P^TDC_EFM_ and buffer F (100 mM KPO4; 0.1 mM pyridoxal-5-phosphate; pH 7.4) for DDC. Protein concentrations were measured spectrophotometrically (Nanodrop 2000, Isogen, De Meern, The Netherlands) using the predicted extinction coefficient and molecular weight from ExPASy ProtParam tool (www.web.expasy.org/protparam/).

### Enzyme kinetics and IC50 curves

Enzyme kinetics were performed in 200 mM potassium acetate buffer containing 0.1 mM PLP (pyridoxal-5-phosphate, P9255, Sigma, The Netherlands) and 10 nM of enzyme at pH 5 for TDC_EFS_ and TDC_EFM_, and pH 4.5 for ^P^TDC_EFM_. Reactions were performed in triplicate using levodopa substrate ranges from 0.5 to 12.5 mM and tyrosine substrate ranges from 0.25 to 2.5 mM. Michaelis–Menten kinetic curves were fitted using GraphPad Prism 7. The human dopa decarboxylase kinetic reactions were performed in 100 mM potassium phosphate buffer at pH 7.4 containing 0.1 mM PLP and 10 nM enzyme concentrations with levodopa substrate ranges from 0.1 to 1.0 mM. Reactions were stopped with 0.7% HClO_4_, filtered and analyzed on the HPLC-ED-system described below. For IC50 curves, the reaction was performed using levodopa as the substrate at concentrations lower or equal to the Km of the decarboxylases (DDC, 0.1 mM; TDC_EFS_ and TDC_EFM_, 1.0 mM; ^P^TDC_EFM_, 0.5 mM) with 10 different concentrations of carbidopa in triplicate (human dopa decarboxylase, 0.005–2.56 µM; bacterial TDCs, 2–1024 µM).

### HPLC-ED analysis and sample preparation

A volume of 1 mL of ice-cold methanol was added to 0.25 mL cell suspensions. Cells and protein precipitates were removed by centrifugation at 20,000 × *g* for 10 min at 4 °C. Supernatant was transferred to a new tube and the methanol fraction was evaporated in a Savant speed-vacuum dryer (SPD131, Fisher Scientific, Landsmeer, The Netherlands) at 60 °C for 1 h 15 min. The aqueous fraction was reconstituted to 1 mL with 0.7% HClO_4_. Samples were filtered and injected into the HPLC system (Jasco AS2059 plus autosampler, Jasco Benelux, Utrecht, The Netherlands; Knauer K-1001 pump, Separations, H. I. Ambacht, The Netherlands; Dionex ED40 electrochemical detector, Dionex, Sunnyvale, USA, with a glassy carbon working electrode (DC amperometry at 1.0 V or 0.8 V, with Ag/AgCl as reference electrode)). Samples were analyzed on a C18 column (Kinetex 5 µM, C18 100 Å, 250 × 4.6 mm, Phenomenex, Utrecht, The Netherlands) using a gradient of water/methanol with 0.1% formic acid (0–10 min, 95−80% H_2_O; 10–20 min, 80–5% H_2_O; 20–23 min 5% H_2_O; 23–31 min 95% H_2_O). Data recording and analysis were performed using Chromeleon software (version 6.8 SR13).

### Bioinformatics

TDC_EFS_ (NCBI accession: EOT87933) was BLASTed against the protein sequences from the NIH HMP data bank using search limits for Entrez Query “43021[BioProject]”. All BLASTp hits were converted to a distance tree using NCBI TreeView (Parameters: Fast Minimum Evolution; Max Seq Difference, 0.9; Distance, Grishin). The tree was exported in Newick format and visualized in iTOL phylogentic display tool (http://itol.embl.de/). Whole genomes or contigs containing the *tdc* cluster were extracted from NCBI and aligned using Mauve multiple genome alignment tool (v 2.4.0, www.darlinglab.org/mauve/mauve.html).

### Incubation experiments of jejunal content

Luminal contents from the jejunum of wild-type Groningen rats (*n* = 5) were suspended in EBB (5% w/v) containing 1 mM levodopa and incubated for 24 h in an anaerobic chamber at 37 °C prior to HPLC-ED analysis (DC amperometry at 0.8 V).

### DNA extraction

DNA was extracted from fecal samples of Parkinson’s patients and jejunal contents of rats using QIAGEN (Cat no. 51504) kit-based DNA isolation^38^ with the following modifications: fecal samples were suspended in 1 mL inhibitEX buffer (1:5 w/v) and transferred to screw-caped tubes containing 0.5 g of 0.1 mm and 3 mm glass beads. Samples were homogenized 3 × 30 sec with 1-minute intervals on ice in a mini bead-beater (Biospec, Bartlesville, USA) three times before proceeding according to manufacturer’s protocol (Isolation of DNA from Stool for Pathogen Detection).

### Quantification of bacterial TDC

To identify bacterial species carrying the *tdc* gene, a broad range of *tdc* genes from various bacterial genera were targeted as previously described^39^ (Supplementary Fig. 5). Quantitative PCR (qPCR) of *tdc* genes was performed on DNA extracted from each fecal sample of Parkinson’s patients and rats’ jejunal content using primers (Dec5f and Dec3r) targeting a 350 bp region of the *tdc* gene. Primers targeting 16S rRNA gene for all bacteria (Eub338 and Eub518) were used as an internal control (Supplementary Table 8). All qPCR experiments were performed in a Bio-Rad CFX96 RT-PCR system (Bio-Rad Laboratories, Veenendaal, The Netherlands) with iQ SYBR Green Supermix (170-8882, Bio-Rad) in triplicate on 20 ng DNA in 10 µL reactions using the manufacturer’s protocol. qPCR was performed using the following parameters: 3 min at 95 °C; 15 sec at 95 °C, 1 min at 58 °C, 40 cycles. A melting curve was determined at the end of each run to verify the specificity of the PCR amplicons. Data analysis was performed using the BioRad software. Ct[DEC] values were corrected with the internal control (Ct[16 s]) and linearized using 2^-(Ct[DEC]-Ct[16 s]) based on the 2^-ΔΔCt method^40^.

### Jejunal and plasma extraction of levodopa metabolites

Levodopa, dopamine, and DOPAC were extracted from each luminal jejunal content and plasma samples of rats using activated alumina powder (199966, Sigma) as previously described^41^ with a few modifications. A volume of 50–200 µl blood plasma was used with 1 µM DHBA (3, 4-dihydroxybenzylamine hydrobromide, 858781, Sigma) as an internal standard. For jejunal luminal content samples, an equal amount of water was added (w/v), and suspensions were vigorously mixed using a vortex. Suspensions were subsequently centrifuged at 20,000× *g* for 10 min at 4°C. A volume of 50–200 µL of supernatant was used for extraction. Samples were adjusted to pH 8.6 with 200–800 µl TE buffer (2.5% EDTA; 1.5 M Tris/HCl pH 8.6) and 5–10 mg of alumina was added. Suspensions were mixed on a roller shaker at room temperature for 15 min and were thereafter centrifuged for 30 s at 20,000× *g* and washed twice with 1 mL of H_2_O by aspiration. Levodopa and its metabolites were eluted using 0.7% HClO_4_ and filtered before injection into the HPLC-ED-system as described above (DC amperometry at 0.8 V).

### Statistical analysis and (non)linear regression models

All statistical tests and (non)linear regression models were performed using GraphPad Prism 7. Statistical tests performed are unpaired *T*-tests, 2-way-ANOVA followed by a Fisher’s LSD test. Specific tests and significance are indicated in the figure legends.

## Supplementary information

Supplementary Information

Reporting Summary

## Data Availability

The authors declare that all the data supporting the findings of this study are available within the paper and its supplementary information files. The sequences of the TDC genes from *E. faecium* W54 TDC_EFM_ and ^P^TDC_EFM_ have been deposited under NCBI accession numbers MH358385, MH358384, respectively. The gene sequence of *E. faecalis* v583 TDC_EFS_ was already available under NCBI accession number EOT87933.
