# Supplementary material for: Gut bacterial tyrosine decarboxylases restrict levels of levodopa in the treatment of Parkinson’s disease
Source: Nat Commun. 2019 Jan 18;10:310. doi: 10.1038/s41467-019-08294-y (PMC6338741; doi:10.1038/s41467-019-08294-y)

Gut bacterial tyrosine decarboxylases restrict levels of levodopa in the treatment of Parkinson's disease

van Kessel *et al*.

Supplementary methods

Screening for *Enterococcus* strains, isolated from healthy subjects and clinical isolates

Enterococcus strains were isolated from fecal or urine samples from clinical setting or from healthy volunteers (aged 2 to 79 years). All samples were collected between January 2008 and 2018 in Beni-Suef City, Egypt. A total of 77 *Enterococcus* spp. were isolated on bile esculin agar and observed microscopically after gram staining. The screening for decarboxylase activity was performed as described previously by Bover-Cid and Holzapfel [1] with levodopa or tyrosine added as a substrate to a final concentration of 1% to screen for production of dopamine and tyramine. All *Enterococcus* strains were spot inoculated on agar plates containing the substrates of interest and on control plates without any substrate. The plates were duplicated, either aerobically or anaerobically at 37°C. Plates were checked daily for 4 days to record the change in the indicator color from yellow to violet, indicative of production of tyramine and dopamine, respectively (**Supplementary Table 1**).

Fecal samples from patients with Parkinson's disease

All study subjects consented to the use of their samples for research. Parkinson's disease was diagnosed according to the UK Brain Bank Criteria. Exclusion criteria for Parkinson's disease subjects: (1) atypical or secondary Parkinsonism, (2) the use of probiotics or antibiotics within three months prior to sample collection, (3) primary gastrointestinal pathology, (4) unstable medical, neurological, or psychiatric illness, (5) low platelet count (<80k), uncorrectable prolonged PT (>15 sec. Solid fecal samples were collected via a home feces collection kit. Study patients were provided with the supplies and instructions for home feces collection using the BD Gaspak EZ Anaerobe Gas Generating Pouch System with Indicator (Ref 260683; Becton, Dickinson and Company, Sparks, MD) in order to minimize the exposure of the feces to high oxygen ambient atmosphere, which may alter the microbiota. Patients were asked to have a bowel movement within 24 hours of their study visit. Patients kept the sealed anaerobic fecal bag in a cold environment, before bringing the anaerobic fecal bag to the hospital. Fecal samples were then immediately stored at -80°C until analysis.

Primers targeting bacterial tyrosine decarboxylase

In order to cover numerous bacterial species harboring the *tdc* gene, degenerate primers, Dec5f and Dec3r, previously designed to target 350bp region of the *tdc* gene from a variety of bacterial genera[2] were used for qPCR. Prior to qPCR experiments a normal PCR test on the genomic DNA *E. faecalis* v583 was performed using Phire Hot Start II DNA Polymerase (F125S, ThermoFisher) using the following PCR parameters: 3 min at 98°C; 15 sec at 98°C, 1 min at 58°C and 1 min at 72°C, for 35 cycles; 5 min at 72°C, to ensure primer specificity.

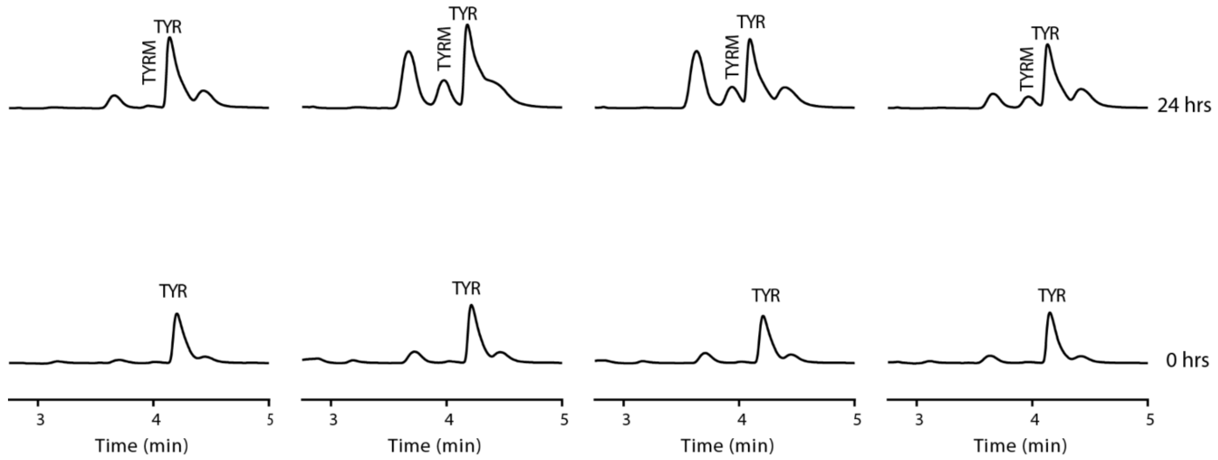

Supplementary Figure 1. Corresponding control samples jejunal content incubation without levodopa. From left to right corresponding control samples, where H_2O was added instead of L-DOPA. Bacterial conversion of tyrosine (TYR) to tyramine (TYRM) during 24 hrs of incubation of jejunal content is visible. No dopamine is produced *de novo* or hydrolyzed from potential conjugated-forms of dopamine in the jejunal-content.

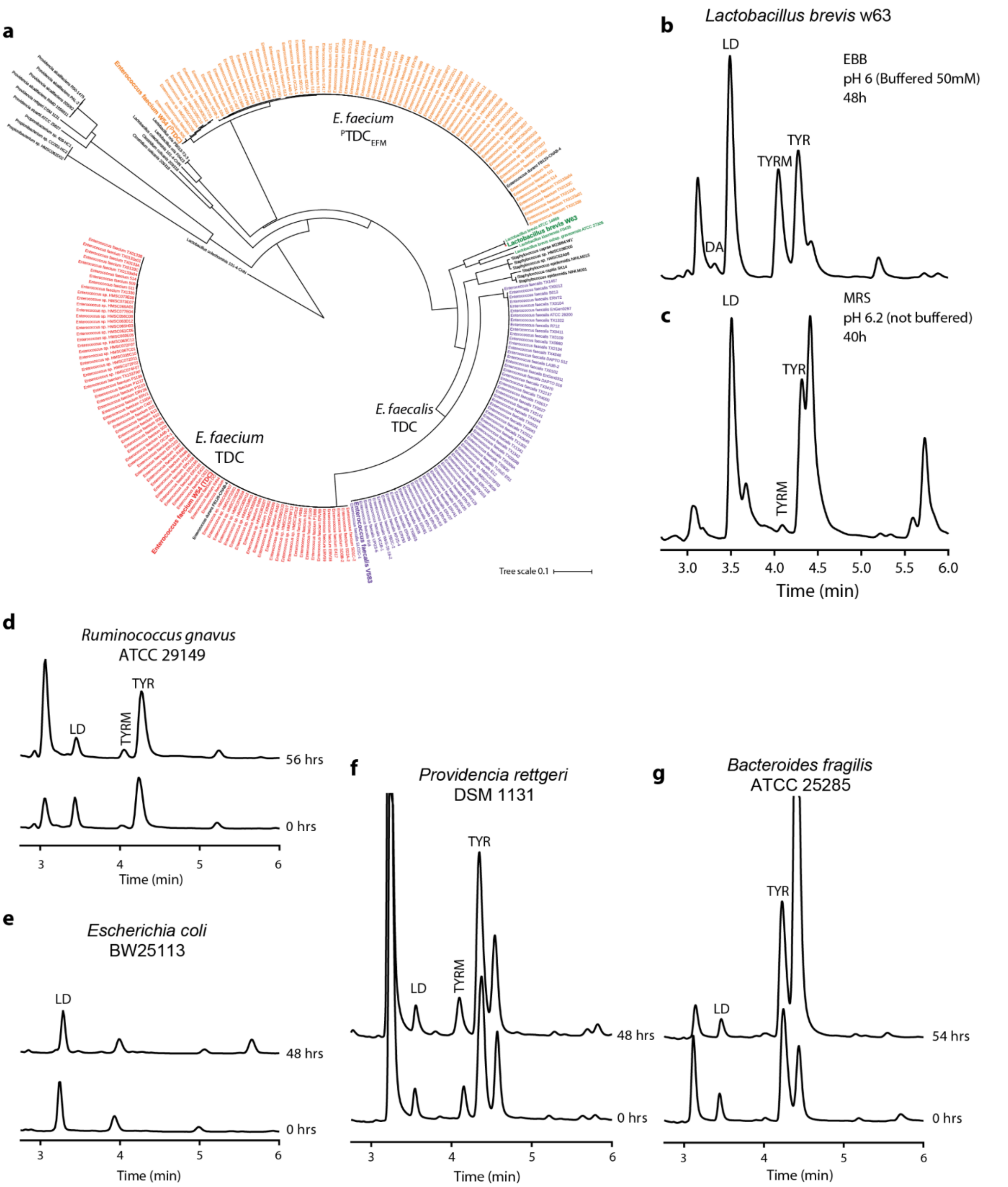

Supplementary Figure 2. Microbiota harboring other PLP-dependent amino acid decarboxylases do not decarboxylate levodopa. (**a**) Phylogenetic analysis of TDC proteins from NIH Human Microbiome Project (HMP) protein database, TDC_{EFS} (EOT87933) was used as query. TDC protein sequences from strains employed in this study are depicted in bold. Live stationary cultures of *L. brevis* grown with levodopa in (**b**) MRS (De Man, Rogosa and Sharpe) or in (**c**) enriched beef broth (EBB) buffered at pH 6.0. (**d-g**) Gut associated bacteria harboring different amino acid decarboxylases, which

were previously identified (**Supplementary Table 2**) were tested for their ability to convert levodopa in live stationary cultures

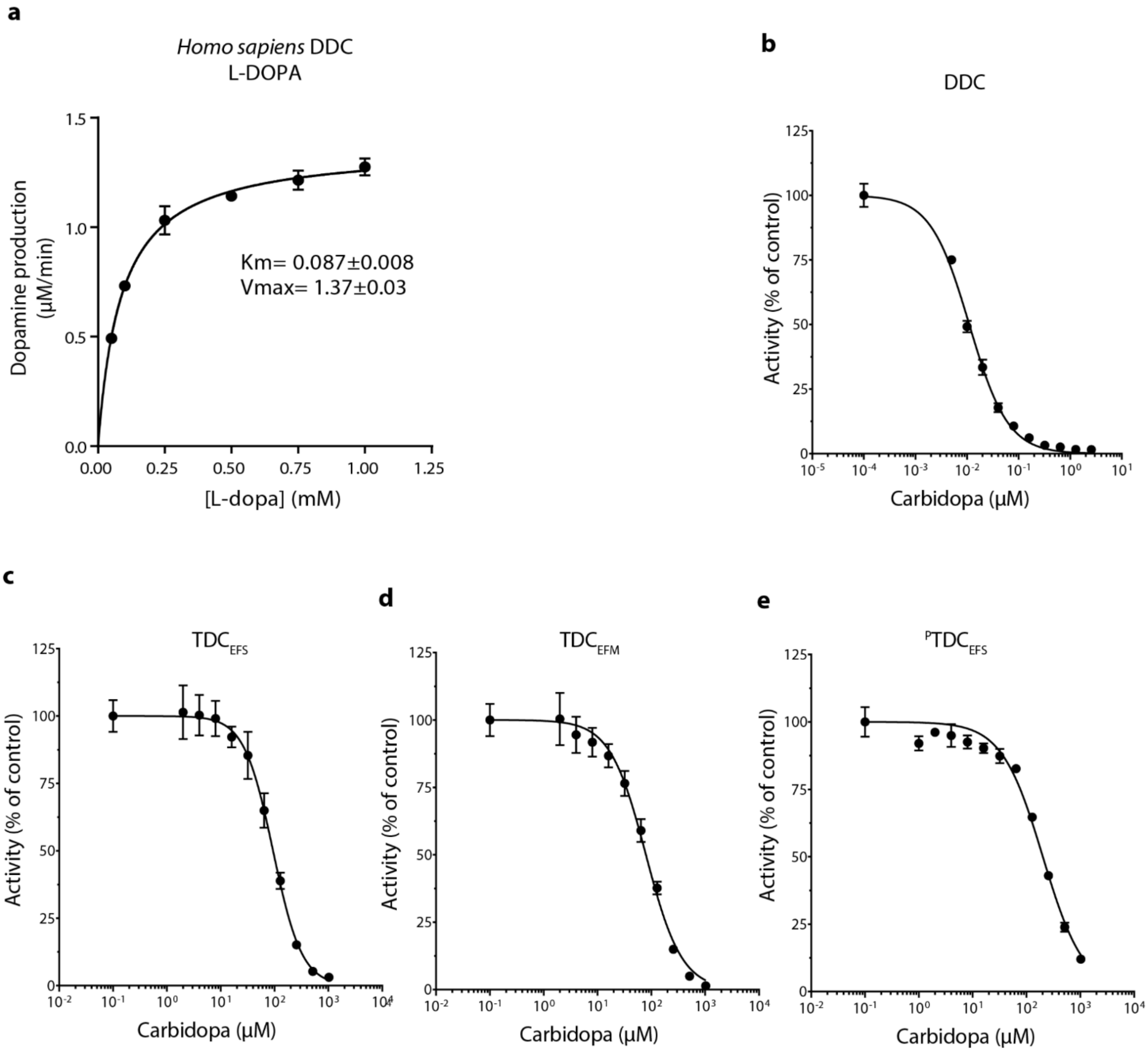

Supplementary Figure 3. IC50 determination for human DOPA decarboxylase and bacterial tyrosine decarboxylases. (**a**) Kinetic curve with levodopa as substrate for human DOPA decarboxylase (DDC) to determine the inhibitory constant for carbidopa. Reactions were performed in triplicate using 10 nM of enzyme in 100 mM PO_4 with 100 μM PLP[3] with levodopa concentrations ranging from 0.1-1.0 mM. The enzyme kinetic parameters were calculated using nonlinear Michaelis-Menten regression model (for further kinetic parameters see **Table 1**). IC50 inhibitory curves using carbidopa as inhibitor for (**b**) DDC (0.005-2.56 μM carbidopa), (**C**) TDC_{EFS} (2-1024 μM carbidopa), (**d**) TDC_{EFM} (2-1024 μM carbidopa), (**e**) $^{P}TDC_{EFM}$ (2-1024 μM carbidiopa). Reactions were performed in triplicate and the parameters were determined by fitting a sigmoidal–curve ([inhibitor] vs. normalized response). Further parameters are listed in **Supplementary Table 3**.

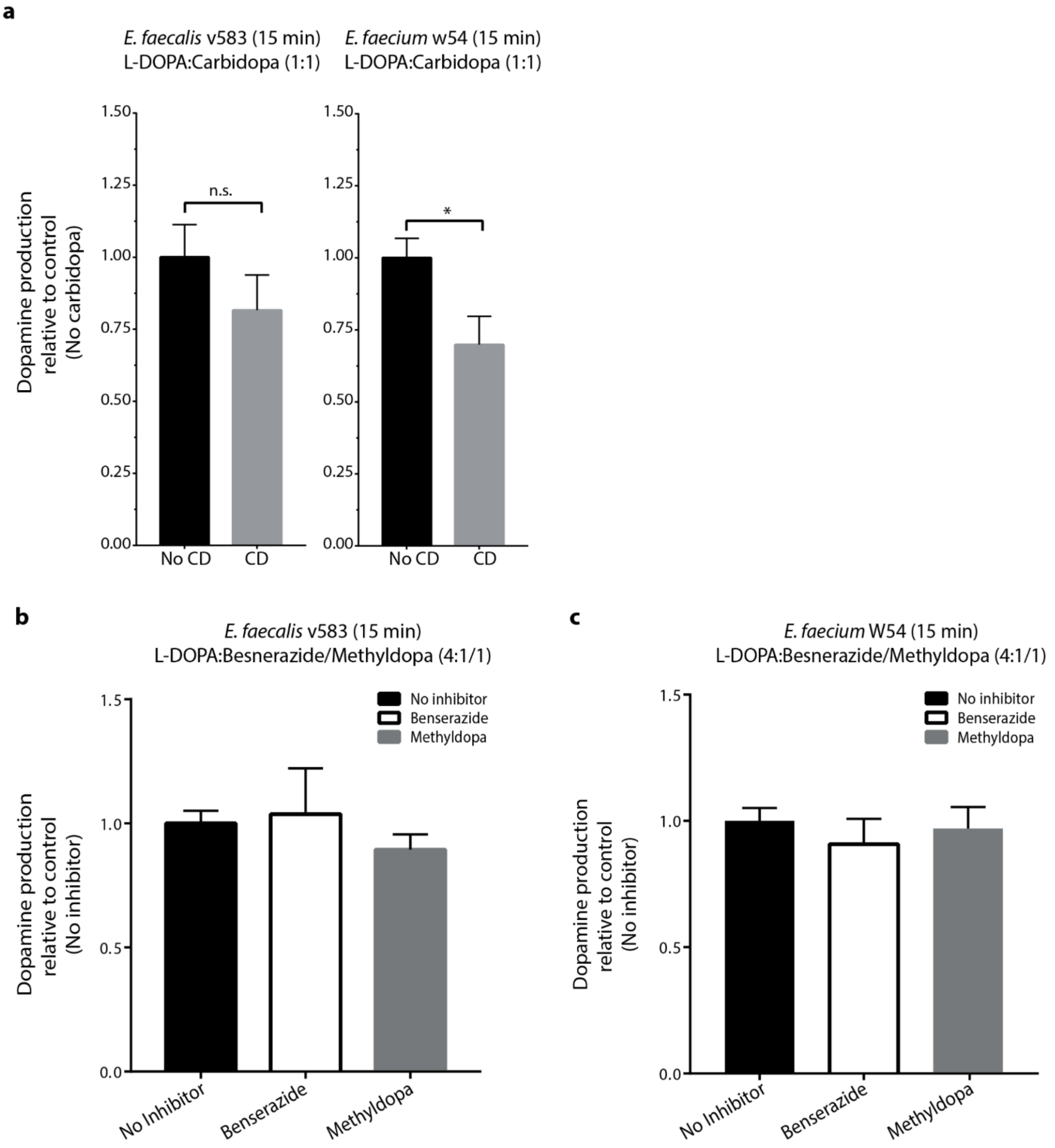

Supplementary Figure 4. Human DOPA decarboxylase inhibitors are ineffective against the decarboxylase activity of live enterococci. Live stationary cultures of *E. faecalis* and *E. faecium* incubated for 15 minutes with (**a**) an equimolar ratio levodopa /carbidopa, (**b, c**) a 6:1 molar ratio (4:1 in weight) of levodopa /benserazide or 4.8:1 molar ratio (4:1 in weight) of levodopa/methyldopa (100/16.7/20,8 μM levodopa /benserazide/methyldopa). Samples were analyzed using HPLC-ED. Bar graphs show levels of dopamine production (relative to control, where no inhibitor was added) with and

without the addition of inhibitor. Error bars represent the SEM and significance was tested using a parametric unpaired T-test (*=$p<0.02$).

a

```
5’-CGTTGTTGGTGTTGTTGGCACNACNGARGARG (Dec5f primer)
    *|||||*|||||*|||||**||||||||||||
5’-TGTTGTCGGTGTAGTTGGTTCTACTGAAGAAGGTGCCGTTGACTCAATCGATAAAAT
TATTGCTTTACGCGATGAATTAATGAAAGACGGTATTTACTATTATGTACACGTTGATGC
TGCTTATGGTGGTTATGGACGTGCCATCTTCTTAGACGAAGACAACAACTTCATCCCTTA
CGAAGATTTACAAGATGTTCACGAAGAATACGGTGTCTTCAAAGAGAAAAAAGAACACAT
TTCAAGAGAAGTGTATGATGCATATAAAGCAATCGAATTAGCAGAATCAGTAACAATTGA
CCCTCATAAAATGGGTTATATCCCTTATTCAGCTGGTGG-3’
          ||||||||||||||*|||||*|||||*||
          ATGGGNTAYRTTCCATATTCTGCTGGCGG-3’ (Dec3r primer)
```

b

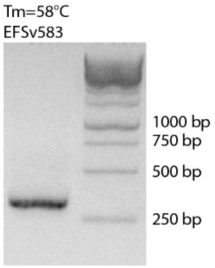

Supplementary Figure 5. Primers (Dec5f and Dec3r) targeting *E. faecalis* v583 *tdc* gene. (**a**) Target of the Dec5f and Dec3r primers[2] are depicted for *Enterococcus faecalis* v583 with (**b**) the corresponding agarose gel of PCR amplification of 336 bp fragment of *tdc*.

Supplementary Figure 6. Identification of conserved *tdc* paralogue protein (TDC_{EFM}) in all *E. faecium* strains analyzed. Identification of conserved *tdc* paralogue protein (TDC_{EFM}) in all *E. faecium* strains analyzed**.** Genome contigs harboring the *tdc* gene cluster of all *E. faecium* strains were extracted from NCBI and aligned using Mauve genome aligner. As comparison, the genome of *E. faecium* W54 is depicted above the alignment results. The paralog TDC from *E faecium* ($^{P}TDC_{EFM}$) is shown in orange. Black boxes indicate the TDC gene in all other strains, white bars indicate the single genes, and colored bars indicate conserved gene clusters. Due to its size, this figure is merged at the end of the supplementary material document.

Supplementary Table 1. ***Enterococcus*** **strains isolated from healthy subjects and clinical enterococcal isolates**

	**Number**	**Sample Type**		**Bile aesculin hydrolysis**	**Levodopa decarboxylation**	**Tyrosine decarboxylation**
	29	Clinical	Stool	29	29	29
	34	Clinical	Urine	34	33	33
	14	Volunteer	Stool	14	10	10
**Total**	**77**			**77**	**72**	**72**

Enterococcus strains isolated from (1) fecal samples (collected from non-hospitalized subjects from clinical labs during their routine check up), (2) urine samples (collected from non-hospitalized patients suffering from urinary tract infection), and (3) healthy volunteers (age 2 to 79 years). All samples were isolated between January 2008-2018 in Beni-Suef City, Egypt. 72 out of 77 isolates were able to decarboxylate levodopa and tyrosine indicating that only 5 isolates are species or strains not encoding for tyrosine decarboxylase.

Supplementary Table 2. List of microbiota harboring other PLP-dependent amino acid decarboxylases tested in this study

Reported tyrosine decarboxylation[4]	Cloned gene[4]	Associated protein accession	Definition	This study (Live bacterial culture)	Protein Identity*	Protein accession
-	BF 0393 (BF9343_0382)	CAH06161	Putative Glutamate decarboxylase	*Bacteroides fragilis* NCTC 9343 (=ATCC 25285)	100.00%	CAH06161
	Not tested	NA	Tyrosine decarboxylase	*Enterococcus faecalis* v583	NA	EOT87933
	Not tested	NA	Tyrosine decarboxylase	*Enterococcus faecium* W54	NA	MH358384, MH358385
-	ECD_03365	ACT45166	Glutamate decarboxylase	*Escherichia coli* BW25113	100.00%	AIN33843
-	LVIS_1847	ABJ64910	Glutamate decarboxylase	*Lactobacillus brevis* W63	NA	
++	LVIS_2213	ABJ65263	Tyrosine decarboxylase	*Lactobacillus brevis* W63	100.00%	MH3583846
-	LVIS_0079	ABJ63253	Glutamate decarboxylase	*Lactobacillus brevis* W63	NA	
+++	PROSTU_02509	EDU59320	putative tyrosine decarboxylase	*Providencia rettgeri* DSM1131	83%	EFE55437
+++	RUMGNA_01526	EDN78222	Tryptophan decarboxylase	*Ruminococcus gnauvs* ATCC29149	100.00%	EDN78222

* Protein identity from strain tested compared to cloned gene Williams et al. [4]. NA, not applicable.

Supplementary Table 3. IC50 curve parameters

	DDC	TDC_{EFS}	TDC_{EFM}	$PTDC_{EFM}$
**[E] (nM)**	10	10	10	10
**[S] (μM)**	100	1000	1000	500
**[Km] (μM)**	87.3	3043.0	7244.0	448.1
**HillSlope**	1.16	1.6	1.3	1.1
**IC50 (μM)**	0.01	93.8	79.2	201.3
**Ki=IC50/(1+([S]/Km))**	0.005	70.6	69.6	95.1
**[IC50]/[s]**	0.01%	9%	8%	40%

The parameters were determined by fitting a sigmoidal–curve ([inhibitor] vs. normalized response) using Graphpad Prism. Reactions were performed in triplicate.

Supplementary Table 4. Sample information Parkinson's patients

ID	*tdc*-gene abundance	Levodopa/carbidopa Tablets per Day	PD Onset (year) - First Diagnosed at Rush University	Disease Duration (years)	Date of Enrollment into Microbiota Research Study at Rush University	UPDRS*	Hy Stage**	Sex	Age	Race	BMI	Medication
Subject 1	1.55E-07	3 Tablets	2010	2	16-5-2012	35	2	Male	63	Caucasian	27.11	No; None
Subject 2	4.30E-07	11 Tablets	2001	11	25-10-2012	30	2.5	Male	50	Caucasian	24.28	No; None
Subject 3	6.48E-08	5 Tablets	2004	8	8-5-2012	36	2	Female	53	Caucasian	22.86	Yes; Naproxyn
Subject 4	8.97E-08	3 Tablets	2008	4	20-8-2012	13	2	Female	58	Caucasian	27.45	No; None
Subject 5	4.01E-07	4 Tablets	1998	14	20-9-2012	26	2	Female	68	Caucasian	23.92	Yes; Aspirin
Subject 6	2.37E-08	3 Tablets	2007	6	4-10-2012	35	2	Male	58	Caucasian	31.44	Yes; Aspirin
Subject 7	6.23E-07	10 Tablets	1991	22	14-6-2012	21	2	Female	57	Caucasian	18.01	No; None
Subject 8	8.69E-08	8 Tablets	2006	6	16-8-2012	27	2	Male	55	Caucasian	23.74	Yes; Aspirin
Subject 9	2.00E-07	6 Tablets	1997	15	18-9-2012	19	2	Male	82	Caucasian	29.12	No; None
Subject 10	2.75E-07	7 Tablets	2002	10	6-12-2012	40	2	Male	74	Asian	28.2	No; None

* Unified Parkinson Disease Rating Scale, ** Hoehn and Yahr Stage, BMI = Body Mass Index. According to medical records, all subjects were on the same levodopa/carbidopa dose thereafter their PD Onset (year) at Rush University Medical Center Neurology Department. Parkinson's disease was diagnosed according to the UK Brain Bank Criteria[5].

Supplementary Table 5. Bivariate correlations

Correlations										
		**L-Dopa Tablets per Day**	**Disease Duration (years)**	**UPDRS**	**Hy Stage**	**Sex**	**Age**	**Race**	**BMI**	**Medica tion**
***tdc*-gene abun dance**	**Pearson Correlation**	0.662*	0.823**	-0.195	0.351	-0.264	0.024	-0.071	-0.652*	-0.401
	**Sig. (2-tailed)**	0.037	0.003	0.589	0.320	0.461	0.949	0.845	0.041	0.251
	**N**	10	10	10	10	10	10	10	10	10

* Correlation is significant at the 0.05 level (2-tailed). ** Correlation is significant at the 0.01 level (2-tailed).

Supplementary Table 6. Bacterial strains and Plasmids used in this study

**Bacterial strains**	**Genotype**	**Reference**
Escherichia coli DH5a	F⁻; *endA1*; *glnV44*; *thi-1*; *recA*; *relA1*; *gyrA96*; *deoR*; *nupG*; *purB20*; φ80d*l acZ*ΔM15; Δ(*lacZYA-argF*)U169; hsdR17($r_K^- m_K^+$); λ⁻	[6]
Escherichia coli BL21 (DE3)	*E. coli* str. B; F⁻; *ompT*; *gal*; *dcm*; *lon*; $hsdS_B$($r_B^- m_B^-$); λ(DE3 [*lacI lacUV5-T7p07 ind1 sam7 nin5*]) ; [$malB^+$]$_{K\text{-}12}$(λ^S)	[7]
Escherichia coli BW25113	$lacI^+$; $rrnB_{T14}$; Δ$lacZ_{WJ16}$; *hsdR*514 Δ$araBAD_{AH33}$; Δ$rhaBAD_{LD78}$; *rph-1*; Δ*(araB–D)567*; Δ*(rhaD–B)568;* Δ*lacZ4787*(::*rrnB-3*)	[8]
E. faecalis v583/ATCC 700802		[9]
E. faecalis ΔTDC	*tyrS*:*nhaC* (Δ*tdcA*)	[10]
Enterococcus faecium W54		Winclove Probiotic B.V.
Lactobacillus brevis W63		Winclove Probiotic B.V.
Ruminococcus gnavus ATCC29149		ATCC
Providencia rettgeri DSM 1131		DSMZ
Bacteroides fragilis ATCC 25285		ATCC
**Plasmids used**	**Gene-insert**	
pET15b		Novagen
pSK11	pET15b-^{P}TDC$_{EFM}$	This study
pSK18	pET15b-TDC$_{EFS}$	This study
pSK22	pET15b-TDC$_{EFM}$	This study
pET15b- OHu25359C	pET15b-DDC cDNA	This study / GenScript

Supplementary Table 7. Constituents of enriched beef broth (EBB) medium used in this study

Component	g/L
Glucose	2.000
NaCl	0.080
K_2HPO_4	5.310
KH_2PO_4	2.650
$NaHCO_3$	0.400
Beef extract	5.000
Yeast extract	3.000
Peptone	0.600
$CaCl_2$	0.008
$MgSO_4$	0.008
Cysteine	0.500
Hemin	0.005
**Vitamin solution (1000x)**	
D-biotin	0.0020
D-Pantothenic acid	0.0100
Ca2.Nicotinamide	0.0050
Vitamin B12	0.0005
Thiamin.HCl	0.0040
Para-aminobenzoic acid	0.0050
Riboflavin	0.0050
Folic acid	0.0020
Pyridoxyal-5-Phosphate	0.0100
Vitamin K1	0.0005
**Trace Elements (1000x)**	
EDTA	1.000
$ZnSO_4.7H_2O$	0.178
$MnSO_4.H_2O$	0.452
$FeSO_4.7H_2O$	0.100
$CoSO_4.7H_2O$	0.181
$CuSO_4.5H_2O$	0.010
H_3BO_3	0.010
$Na_2MoO_4.2H_2O$	0.010
$NiSO_4.6H_2O$	0.111

The KPO_4 solution is buffered at 50 mM pH7.

Supplementary Table 8. Primer sequences used in this study

**Primers**	**5'-Sequence-'3**	**Target**
sk075	AGGAGGCTCGAGAAAGATATGGATATCAAGGCCG	*E. feacium* W54 $^{P}TDC_{EFM}$
sk076	AGGAGGGGATCCCCAGTATCACCGAAACATCC	*E. feacium* W54 $^{P}TDC_{EFM}$
sk153	AGGAGGCTCGAGAAAAACGAAAAATTAGCAAAAGGCG	*E. faecalis* v583 TDC_{EFS}
sk154	AGCAGAGGATCCCAATCAGACGAACGTTCCCTC	*E. faecalis* v583 TDC_{EFS}
sk171	AGAGAGCTCGAGAGTGAATCATTGTCGAAAG	*E. feacium* W54 TDC_{EFM}
sk172	ATATATGGATCCCCAAACATGCGTCAGAAACAG	*E. feacium* W54 TDC_{EFM}
**qPCR**	**5'-Sequence-'3**	**Reference**
DEC5f	CGTTGTTGGTGTTGTTGGCACNACNGARGARG	[2]
DEC3r	CCGCCAGCAGAATATGGAAYRTANCCCAT	[2]
Eub338	ACTCCTACGGGAGGCAGCAG	[11]
Eub518	ATTACCGCGGCTGCTGG	[11]

Underlined sequences represent restriction sites used.

References

1 Bover-Cid, S. & Holzapfel, W. H. Improved screening procedure for biogenic amine production by lactic acid bacteria. *International journal of food microbiology* **53**, 33-41 (1999).

2 Torriani, S. *et al.* Rapid detection and quantification of tyrosine decarboxylase gene (tdc) and its expression in gram-positive bacteria associated with fermented foods using PCR-based methods. *Journal of food protection* **71**, 93-101 (2008).

3 Montioli, R., Cellini, B. & Borri Voltattorni, C. Molecular insights into the pathogenicity of variants associated with the aromatic amino acid decarboxylase deficiency. *Journal of inherited metabolic disease* **34**, 1213-1224, (2011).

4 Williams, B. B. *et al.* Discovery and characterization of gut microbiota decarboxylases that can produce the neurotransmitter tryptamine. *Cell host & microbe* **16**, 495-503, (2014).

5 Keshavarzian, A. *et al.* Colonic bacterial composition in Parkinson's disease. *Movement disorders : official journal of the Movement Disorder Society* **30**, 1351-1360, (2015).

6 Grant, S. G., Jessee, J., Bloom, F. R. & Hanahan, D. Differential plasmid rescue from transgenic mouse DNAs into Escherichia coli methylation-restriction mutants. *Proceedings of the National Academy of Sciences of the United States of America* **87**, 4645-4649 (1990).

7 Wood, W. B. Host specificity of DNA produced by Escherichia coli: bacterial mutations affecting the restriction and modification of DNA. *Journal of molecular biology* **16**, 118-133 (1966).

8 Datsenko, K. A. & Wanner, B. L. One-step inactivation of chromosomal genes in Escherichia coli K-12 using PCR products. *Proceedings of the National Academy of Sciences of the United States of America* **97**, 6640-6645, (2000).

9 Sahm, D. F. *et al.* In vitro susceptibility studies of vancomycin-resistant Enterococcus faecalis. *Antimicrobial agents and chemotherapy* **33**, 1588-1591 (1989).

10 Perez, M. *et al.* Tyramine biosynthesis is transcriptionally induced at low pH and improves the fitness of Enterococcus faecalis in acidic environments. *Applied microbiology and biotechnology* **99**, 3547-3558, (2015).

11 Fierer, N., Jackson, J. A., Vilgalys, R. & Jackson, R. B. Assessment of Soil Microbial Community Structure by Use of Taxon-Specific Quantitative PCR Assays. Appl. Environ. Microbiol. **71**, 4117–4120 (2005).

Supplementary Figure 6

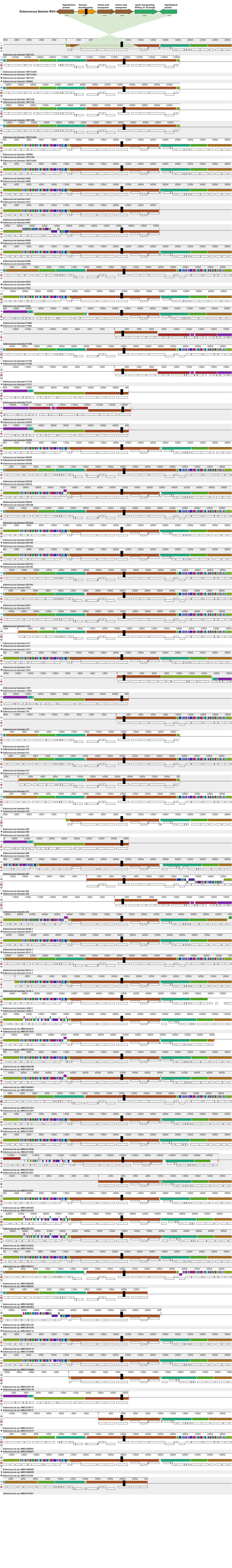

Supplement: Supplementary file 1 — Supplementary Information [file 41467_2019_8294_MOESM1_ESM.pdf]
